# Improving the Stability of Transfersome Systems by Co-Encapsulating Components of Varying Hydrophobicity

**DOI:** 10.3390/pharmaceutics18070859

**Published:** 2026-07-14

**Authors:** Xin Shen, Mian Zhao, Muhammad Hamza, Wen-Ting Wu, Jing Liu, Zi-Lu Guo, Zhi-Yu Guan, Zhe Li, Wei-Feng Zhu

**Affiliations:** 1 College of Pharmacy, Jiangxi University of Chinese Medicine, Nanchang 330004, China; 15270813951@163.com (X.S.); 19314677641@163.com (M.Z.); m.hamza.nano@gmail.com (M.H.); 2 Key Laboratory of Modern Chinese Medicine Preparations Ministry of Education, Traditional Chinese Medicine Pharmaceutical Technology Collaborative Innovation Institute, Jiangxi University of Chinese Medicine, Nanchang 330004, China; 3 Key Laboratory of Modern Chinese Medicine Preparations Ministry of Education, Jiangxi University of Chinese Medicine, Nanchang 330004, China; liujing860828@163.com (J.L.); 15970989223@163.com (Z.-L.G.); adlaiguan@126.com (Z.-Y.G.); lizhezd@163.com (Z.L.); zwf0322@126.com (W.-F.Z.)

**Keywords:** transfersomes, Pueraria total flavones, tanshinone, co-loading, stability

## Abstract

**Background**: As a novel drug delivery vehicle, transfersomes (TFSs) have great deformability, good biocompatibility, and biodegradability, which can significantly improve drug delivery efficiency. However, their application is limited by their poor stability and vulnerability to leaks. **Methods**: This paper prepared and assessed the stability of transfersomes co-loaded with Pueraria total flavonoids (PTFs) and tanshinone (TAN), known as PTF/TAN-TFSs, in order to overcome this constraint. **Results**: The findings showed that the co-loaded system (PTF/TAN-TFSs) had better structural stability than the single-loaded systems (PTF-TFSs and TAN-TFSs), with drug leakage rates decreased by roughly 5% and 7%, respectively, and a more uniform size distribution with a particle size of 44.52 ± 0.47 nm and a PDI of 0.22, while the single-loaded systems had PDI values larger than 0.3. The co-loaded system showed a better sustained-release profile than the suspension with a 15.28% decrease in the maximum cumulative release of the poorly soluble component Tan IIA. Additionally, the co-loaded system demonstrated enhanced solubilization capability, making the poorly soluble component TAN more soluble by 89.52 times compared to the free medication and by 1.97 times compared to the single-loaded system. Furthermore, the co-loaded system significantly improved the intestinal absorption of Tan IIA, increasing the Papp and Ka values in the ileum segment by 2.02-fold and 1.80-fold, respectively. **Conclusions**: The stability of the system was successfully improved by co-loading many components with varying hydrophobicities. This method provides a fresh way to enhance transferosome stability, broadening their application in the field of drug delivery.

## 1. Introduction

Transfersomes (TFSs), as second-generation ultra-deformable elastic liposomes, are now recognized as a research hotspot in the field of nanomedicine drug delivery due to their unique highly flexible membrane structure and excellent ability to penetrate biological barriers [1]. Unlike conventional liposomes, transfersomes can pass through biological membrane pores (like the intercellular spaces of the stratum corneum and intestinal epithelial cells) that are significantly smaller than their own diameter while retaining structural integrity because they are highly deformable due to the incorporation of edge activators (such as sodium cholate and Tween 80) into the phospholipid bilayer [2,3,4]. When it comes to the transport of medications like insulin, ketoprofen, and quercetin, transfersomes surpass traditional liposomes in terms of penetration efficiency and therapeutic efficacy, according to numerous studies [5,6,7]. Although transfersomes hold promising potential in the field of drug delivery, they still suffer from inherent limitations in practical applications, the most prominent of which is their poor intrinsic stability both in vitro and in vivo [8]. The transferosome membrane’s high surfactant ratio and low cholesterol concentration cause loose bilayer packing and excessive fluidity, which poses a serious stability problem. Particularly, this shows up as significant drug leakage during storage, vesicle aggregation, fusion, and a sharp increase in particle size. These problems raise production costs in addition to reducing therapeutic efficacy [9].

Researchers have mostly used optimization techniques, such as changing formulas, streamlining preparation procedures, and structurally altering transfersomes, to increase their stability. According to one study, the compactness of the vesicle membrane might be somewhat improved by changing the type of surfactant while maintaining the vesicle matrix materials [10,11]. Khan et al. showed that transfersomes made with Tween 80 as the surfactant were much more stable than those made with Span 80 and Span 20. This formulation successfully improved the vesicles’ membrane compactness and physical stability, decreased drug leakage and vesicle aggregation, and maintained favorable deformability [11]. Additionally, it has been demonstrated that changing the formulation’s phase state efficiently prevents vesicle aggregation, which is another effective way to increase the formulation’s long-term storage stability [12,13]. Lu et al. prepared Panax notoginseng saponins-loaded transfersomes using a lyophilization process with a trehalose–sucrose composite system as a cryoprotectant. Compared with liquid transfersomes, the lyophilized formulation exhibited significantly improved storage stability. Moreover, after reconstitution, its transdermal performance remained comparable to that of the freshly prepared liquid formulation, thereby fundamentally overcoming the inherent stability issues of liquid transfersomes, such as aggregation, sedimentation, and oxidative degradation [13]. Furthermore, according to certain research, the PEGylation of transfersomes may form a hydrophilic steric barrier on the vesicle surface, inhibiting membrane fusion and particle aggregation [14,15]. For instance, Deng et al. showed that DSPE-mPEG2000-modified transfersomes exhibited higher physical storage stability and successfully suppressed particle aggregation and drug leakage even after three months of storage at 4 °C and 25 °C when compared to unmodified conventional transfersomes [15]. Although the aforementioned tactics have made some headway, they all seek to increase stability by altering the delivery system’s process conditions, ignoring the possible impact of component interactions to stability.

Notably, recent studies have revealed that the co-encapsulation of two or more active ingredients can improve the ordering of lipid bilayers through intermolecular interactions, providing important theoretical support for this paper. The strongly hydrophobic component is stably anchored in the hydrophobic inner core of the bilayer, while the weakly hydrophobic component fills the interstitial gap of the lipid membrane. This complementary embedding mode optimizes the compactness and uniformity of the lipid bilayer structure [16,17]. Additionally, our earlier research has shown that co-loading components with various hydrophobicities in a self-assembled system can improve the aggregates’ structural compactness and, thus, the formulation’s overall physical stability [18]. Thus, by co-encapsulating suitable active substances, this paper suggests a method to improve the stability of transfersomes. For instance, Puerarin total flavonoids (PTFs) and tanshinone (TAN), the active ingredients of the traditional Chinese medicine duo Pueraria lobata–Salvia miltiorrhiza, were chosen as models of weakly and strongly hydrophobic compounds, respectively, and co-loaded into transfersomes. To assess the impact of drug co-loading on improving transfersome stability and intestinal drug absorption, the physicochemical characteristics, drug leakage, in vitro release, and in vivo performance (including intestinal absorption, degradation, and distribution) of the co-loaded transfersomes (PTF/TAN-TFSs) were compared with those of single-loaded transfersomes (PTF-TFSs and TAN-TFSs). This paper provides a novel strategy for enhancing the colloidal stability of transferosomes. Meanwhile, the co-encapsulation approach efficiently addresses the poor aqueous solubility of hydrophobic drugs, laying a solid scientific foundation for the rational design of modern vesicular drug delivery systems.

## 2. Materials and Methods

### 2.1. Materials

Sodium deoxycholate was purchased from Yien Chemical Technology Co., Ltd. (Shanghai, China). Egg yolk lecithin, puerarin reference standard, and phosphotungstic acid negative staining solution were purchased from Solarbio Technology Co., Ltd. (Beijing, China). Puerarin isoflavone fractions were purchased from Shennong Biotechnology Co., Ltd. (Xi’an, China). Tanshinone fractions were purchased from Guangyi Biotechnology Co., Ltd. (Chengdu, China). 3′-Hydroxypuerarin, 3′-methoxypuerarin, and Tanshinone IIA reference standards were purchased from Pufei De Biotechnology Co., Ltd. (Chengdu, China). Methanol, phosphoric acid, potassium dihydrogen phosphate, sodium dihydrogen phosphate, sodium hydroxide, dichloromethane, anhydrous calcium chloride, sodium chloride, and sodium bicarbonate chloride (analytical grade) were purchased from Science Co., Ltd. (Xilong, China). Acetonitrile (chromatography grade) was purchased from Fudun Science and Technology Co., Ltd. (Wuhan, China). A P2 probe [19,20] was provided by the Wu Wei research group at Fudan University; Ethyl carbamate (analytical grade) was purchased from the Aladdin Biochemical Technology Co., Ltd. (Shanghai, China). Glucose and magnesium chloride (analytical grade) were purchased from the Damiao Chemical Reagent Factory (Tianjin, China).

BALB/c mice, SPF grade, male, weighing 20 ± 2 g, purchased from Sibeifu (Beijing, China) Biotechnology Co., Ltd., Production License No.: SCXK(Jing)22024-0001. Mouse husbandry and use comply with China’s Regulations on the Management of Laboratory Animals. Experimental animals underwent one week of acclimatization with free access to food and water. Additionally, SD rats, SPF grade, male, weighing 200–250 g, were purchased from Henan Skebes Biotechnology Co., Ltd. (Henan, China), Production License No.: SCXK (Henan) 2022-0002. Rat husbandry and use complied with China’s Regulations on the Management of Laboratory Animals. Experimental animals underwent a one-week acclimatization period with free access to food and water.

### 2.2. Evaluation of the Physicochemical Properties of the Drug-Loaded TFSs

#### 2.2.1. Preparation of the Drug-Loaded TFSs

Thin-film hydration was used to create a number of TFSs loaded with various hydrophobic single and multi-component medications [21,22]. We precisely weighed 375 mg of carrier material at a fixed mass ratio (EPC: SDC = 2:1) [23]. Then, we filled a 100 mL round-bottom flask with drugs (PTF 50 mg, TAN 1.5 mg). To ensure total dissolution, we added 20 milliliters of methanol. To create a film, we removed the methanol with rotary evaporation at 40 °C and 50 rpm/min. To form a dry, translucent membrane scaffold, we vacuum dried the medication at ambient temperature for the entire night. To obtain single-component PTF-TFSs and TAN-TFSs as well as the co-loaded PTF/TAN-TFSs, we added 10 mL of ultrapure water to the film, shook it for 10 min to hydrate it, and then filtered it through a 0.22 µm cellulose acetate membrane. We used the same method to prepare KB-TFSs without drugs.

#### 2.2.2. Measurement of the Particle Size of the Drug-Loaded TFSs

A Malvern Zetasizer Nano-ZS was used to measure the particle sizes of KB-TFSs, single-component-loaded PTF-TFSs, TAN-TFSs, and multi-component-loaded PTF/TAN-TFSs. The following parameters were used for the measurement: temperature 25 °C, viscosity 0.8872 cP, refractive index 1.333, and wavelength 632.8 nm.

#### 2.2.3. Appearance and Morphological Characterization of Component-Loaded TFSs

The prepared component-loaded TFSs were individually transferred into 10 mL transparent sample vials, and their clarity was visually compared with that of suspensions containing each single component and the mixed components. A 10 μL aliquot of the drug-loaded TFSs, diluted tenfold, was deposited onto a copper grid coated with a supporting film. After standing for 3 min, the excess sample was removed with filter paper, which was followed by the addition of 10 μL of 2.0% (*w*/*v*) phosphotungstic acid staining solution. The samples were air-dried at room temperature for 24 h, and the morphology of the TFSs was then observed using a transmission electron microscope at an operating voltage of 80 kV.

#### 2.2.4. Evaluation of the Solubilization Effect of TFSs on the Components

One co-loaded TFS (PTF/TAN-TFSs) and two single-component-loaded TFSs (PTF-TFSs and TAN-TFSs) were built using the method described under “Preparation of drug-loaded TFSs.” After hydration, the supernatant was collected and run through a 0.22 μm pore cellulose acetate membrane. The PTF and Tan IIA concentrations in the TFS systems were then determined using high-performance liquid chromatography.

#### 2.2.5. High-Performance Liquid Chromatography Conditions

PUE, 3′-HPUE, and 3′-MPUE: Column: ACE Excel 5 C18 column (5 μm, 250 × 4.6 mm); mobile phase: acetonitrile–0.1% aqueous phosphoric acid solution; gradient elution: 0–5 min, 5–15% acetonitrile; 5–10 min, 15–14% acetonitrile; 10–15 min, 14% acetonitrile; flow rate: 1.0 mL/min; detection wavelength: 254 nm; column temperature: 30 °C; injection volume: 5 μL.

TanIIA: column: ACE Excel 5 C18 column (5 μm, 250 × 4.6 mm); mobile phase: acetonitrile–0.1% aqueous phosphoric acid solution (80:20), isocratic elution; flow rate: 1.0 mL/min; detection wavelength: 270 nm; column temperature: 30 °C; injection volume: 20 μL.

### 2.3. In Vitro Stability Evaluation of Component-Loaded TFSs

#### 2.3.1. Particle Size Tracking of Loaded TFSs

To evaluate the drug-loading stability of TFSs, the generated TFS systems loaded with one or more components were maintained at 37 °C with constant humidity and temperature. The particle sizes of each loaded system were measured using a Malvern laser particle size analyzer after 0, 12, 24, 36, and 48 h as well as after a week. The changes in particle size over time were recorded.

#### 2.3.2. Determination of the Drug Leakage Rate of Component-Loaded TFSs

The drug leakage behavior of each hydrophobic component in TFS solutions was assessed in order to look at how various hydrophobic components affected drug-loading stability. The samples were kept in a chamber with continuous humidity and temperature. The supernatant was collected and filtered at 0, 12, 24, 36, and 48 h as well as after a week. A 10 mL volumetric flask was then filled with 0.5 mL of the filtrate, sonicated with methanol, and diluted to volume. The drug concentration was then ascertained by passing the mixture through an organic membrane filter with a pore size of 0.22 μm. Tan IIA and PUE leakage rates in various systems were computed appropriately.

$$leakage\;rate\left(\%\right)=\frac{\left(Drug\;Quality\;in\;TFSs-Drug\;concentration\;in\;TFSs\;after\;48\;h\right)}{Drug\;Quality\;in\;TFSs}$$

### 2.4. In Vitro Release Study of Component-Loaded TFSs

#### 2.4.1. Preparation of Release Medium

Making a pH 6.8 phosphate buffer: We weighed 0.896 g of sodium hydroxide and 6.805 g of potassium dihydrogen phosphate precisely. Then, we diluted them to 1000 mL after dissolving in ultrapure water and mixed well. Afterwards, we used a sodium hydroxide solution to bring the pH down to 6.8.

Screening of solubilizer ratios in release medium: Due to the poor water solubility of TanIIA in the TAN component [24], specific ratios of Tween 80 and HP-β-cyclodextrin solubilizers needed to be added to the release medium to enhance its solubility and meet leaching conditions. Solutions of the two solubilizing agents at concentrations of 0.1%, 0.5%, and 1% were prepared using phosphate buffer at pH 6.8 to determine the optimal solubilizing agent ratio. Excessive amounts of TAN components were added to screw-cap centrifuge tubes containing 10 mL of release medium with different solubilizer ratios. The tubes were placed in a 37 °C air-controlled shaking incubator and shaken at 100 r/min for 24 h until dissolution equilibrium was reached. We centrifuge at 10,000 rpm for 10 min. Then, we took an appropriate amount of supernatant, filtered it through a 0.22 μm microporous membrane, injected it, and calculated the solubility of Tan IIA in different release media.

#### 2.4.2. In Vitro Release

The reverse dialysis method was used to quantify TAN in order to better attain the leakage conditions [25,26,27], and the PTF was measured using the forward dialysis method. As per the formulation, we prepared three samples with equal drug content for each of the drug delivery system groups (PTF/TAN-TFSs, PTF-TFSs, TAN-TFSs) and suspension groups (PTF/TAN-Suspension, PTF-Suspension, TAN-Suspension). Dialysis in reverse: We removed precisely one milliliter of release medium (pH 6.8 phosphate buffer with 1% Tween 80) and transferred it into eight identical-sized and shaped dialysis bags. After securely tying the ends of the dialysis bags, we completely submerged them in 100 milliliters of release medium to allow for 12 h of equilibration. Next, we carefully removed 5 mL of the TAN group sample and put it on the outside of the dialysis bags. We kept the temperature of the release medium at 37 ± 0.5 °C while rotating at 100 revolutions per minute. One dialysis bag was removed at 2, 4, 6, 8, 10, 12, 24, and 36 h. One milliliter of internal medium was aspirated, and the same volume of fresh medium at the same temperature was added right away. After the sample had been passed through a 0.22 μm microporous membrane, we injected it. We determined the concentration of TanIIA and PUE as well as the cumulative release quantity Q (%). The cumulative release degree is calculated using the following formula:

$$Q\%=\frac{v_{e}\sum_{0}^{n-1}C_{n}+vC_{n}}{m}\times 100\%$$

Q is the cumulative release, ve is the sampling volume, $C_{n}$ is the drug concentration at the nth time point, m is the drug content in the dialysis bag, and v is the volume of the release medium.

### 2.5. In Vivo Intestinal Absorption Study of Delivery Systems Loaded with Different Hydrophobic Components

#### 2.5.1. Preparation of Perfusion Fluid

Krebs–Ringer’s Solution (K-R Solution) is made by weighing 7.890 g of NaCl, 0.359 g of KCl, 0.029 g of MgCl_2_, 0.329 g of NaH_2_PO_4_, 0.37 g of CaCl_2_, and 1.400 g of glucose in 700 mL of distilled water. We mixed the solution well before adding 1.379 g of NaHCO3, using ultrasonication to dissolve it, and then diluting it with 1 L of distilled water. To finish, we thoroughly mixed the solution again. (The test solution needs to be ready right away. To avoid precipitation, we ensured that the calcium chloride was completely dissolved before adding additional ingredients.)

Blank Intestinal Perfusion Fluid Preparation: We used a suitable volume of K-R solution and adhered to the experimental protocol described in the “In vivo single-pass intestinal perfusion experiment in rats” section. After gathering the effluent liquid and centrifuging it to extract the supernatant, the blank intestinal perfusion fluid was prepared.

Preparation of TFSs Perfusion Solutions: We followed the experimental procedure under “Preparation of the drug-loaded TFSs” to obtain TFSs loaded with components. Subsequently, we added 100 mL of K-R solution and hydrated it for 10 min to prepare perfusion solutions for single-component PTF-TFSs, TAN-TFSs, and co-loaded PTF/TAN-TFSs.

Drug Suspension Perfusion Solution: To make PTF/TAN Suspension, we precisely weighed 50 mg PTF and 3 mg TAN, dissolved them in K-R solution, and adjusted the solution to 100 mL. Then, we used the same procedure to prepare the PTF and TAN suspension perfusion solutions.

#### 2.5.2. In Vivo Single-Pass Intestinal Perfusion Experiment in Rats

SD rats [SYXK(Gan)2017-0004] were fasted for 12 h (with free access to water) and divided into 5 groups of 3 rats each. An intraperitoneal injection of 5 mL/kg of a 20% urethane solution was given. A 3–4 cm midline abdominal incision was performed after anesthesia. A 10 cm section of the jejunum (15–25 cm distal to the pylorus) and a 10 cm section of the ileum (20–30 cm proximal to the cecum) were separated using normal techniques after the rat’s digestive tract and mesentery were dissected. We returned to the abdominal cavity after ligating both ends and used saline that had been preheated to 37 °C to flush and empty the colon. We maintained body temperature under an infrared lamp while covering the wound with moistened gauze. For 30 min, we perfused with K-R solution at 37 °C and 0.2 mL/min. After 30 min of equilibration, we switched to the appropriate concentration of drug-containing intestinal perfusion solution at 37 °C and 0.2 mL/min. After that, we swapped out the EP tubes with known masses of perfusion and collection fluids every 15 min to obtain eight samples over the course of 120 min. We weighed the infused and collected fluid masses after cooling the effluent. To determine the densities of each infused and recovered medication solution, we aspirated one milliliter. After the experiment was complete, we removed the intestine segment, measured its circumference and length three times, and then computed the cross-sectional radius (r).

#### 2.5.3. Processing of Perfusion Samples

We filled a 1.5 mL centrifuge tube with 0.5 mL of each intestinal perfusion fluid sample. To ensure complete mixing, we added 0.5 mL of methanol, vortexed for 30 s, centrifuged at 12,000 rpm for 10 min, collected the filtrate, filtered the supernatant through a 0.22 μm microporous membrane, and determined the PUE and TanIIA content in the perfusion fluid samples from the jejunum and ileum at each time point.

#### 2.5.4. Data Processing

The apparent permeability coefficient (Papp) and absorption rate constant (Ka) of a drug are calculated using the gravimetric method, according to the following formulas:

$$P_{app}=-Q\times \ln(\frac{C_{out}}{C_{in}}\times \frac{V_{out}}{V_{in}})\times \frac{1}{2\pi rL}$$

$$K_{a}=(1-\frac{C_{out}}{C_{in}}\times \frac{V_{out}}{V_{in}})\times \frac{Q}{\pi r^{2}L}$$

L and *r* represent the length (cm) and cross-sectional radius (cm) of the perfused intestinal segment, respectively; $C_{in}$ and $C_{out}$ represent the drug concentrations (g/mL) in the perfusion solution and the collected solution, respectively; Q represents the flow rate (0.2 mL/min) of the intestinal perfusion solution.

### 2.6. In Vivo Degradation and Distribution Study of Oral Delivery System Components of TFSs Using ACQ Probe P2 Tracer

#### 2.6.1. Preparation of Solutions

P2-labeled TFS preparation: To create a 100 μg/mL stock solution, we weighed out 1 milligram of the P2 probe, dissolved it in acetonitrile, and diluted it to 10 mL in a volumetric flask. We put 200 μL of the stock solution into a 100 mL round-bottom flask, let it evaporate under nitrogen, and then added the prescription amount of medication along with 375 mg of carrier material with a set mass ratio (EPC:SDC = 2:1). To create a thin film, we dissolved it in methanol and used a rotary evaporator with lowered pressure at 40 °C and 50 rpm. To create P2-labeled PTF-TFSs, TAN-TFSs, and PTF/TAN-TFSs, we hydrated the film with ultrapure water following overnight storage.

Preparation of P2 Water Quenching Solution: We dissolved the P2 probe in acetonitrile to a concentration of 100 μg/mL. We took 1 mL of this solution and mixed it with 20 mL of ultrapure water. Then, we removed the acetonitrile by rotary evaporation at 40 °C and 50 rpm and added ultrapure water to achieve the same concentration as the probe in P2-TFSs. We designated P as the control group to evaluate the potential interference in quantification caused by fluorescence re-ignition.

#### 2.6.2. In Vivo Degradation and Distribution Behavior of P2-TFSs After Oral Administration

The in vivo real-time imaging of P2-labeled TFSs was performed following the methods described in the relevant literature [28,29]. Twelve male BALB/c mice (fasted for 12 h prior to the experiment with free access to water, and abdominal fur removed using depilatory cream) were randomly divided into four groups (P2-KB-TFSs, P2-PTF/TAN-TFSs, P2-PTF-TFSs, and P2-TAN-TFSs) with three mice in each group. A P2 solution quenched with water was used as the control group. Baseline images were collected before administration. Each group was gavaged with 0.2 mL of the test formulation, and the fluorescence intensity was adjusted slightly to maintain uniformity across groups. At the time points of 0.5, 1, 2, 4, 6, 8, 12, 24, and 36 h after administration, mice were anesthetized with isoflurane, and P2 signals were collected using the FOBI system. Near-infrared fluorescence intensity was recorded to compare the differences in in vivo degradation and distribution after oral administration.

#### 2.6.3. Investigation of Gastrointestinal Distribution of P2-TFSs After Oral Administration

To further investigate the distribution and absorption of P2-TFSs in the gastrointestinal tract after oral administration, three mice were euthanized at each of the specified time points mentioned above. The gastrointestinal tract was carefully separated, rinsed with saline, and excess moisture was absorbed with filter paper. The tissue was placed on a black plastic board, and near-infrared fluorescence signal images were captured using the FOBI system [30,31] equipped with a near-infrared filter set.

#### 2.6.4. Fluorescence Monitoring of P2-TFSs in Blood

About 0.5 mL of blood was drawn from the orbital venous plexus of mice at predetermined intervals (0.5, 1, 2, 4, 6, 8, 12, 24, and 36 h) and put in heparinized tubes. After centrifuging the samples for ten minutes at 4 °C at 3000 rpm, 100 µL of plasma was recovered and placed on a 96-well plate. The Region of Interest (ROI) method-based FOBI system was used to quantify fluorescence intensity right away [32].

## 3. Results and Discussion

### 3.1. Study on the Physicochemical Properties of Component-Loaded TFSs

#### 3.1.1. Characterization of the Appearance and Morphology of TFSs Co-Loaded with Different Hydrophobic Components

**Figure 1:**
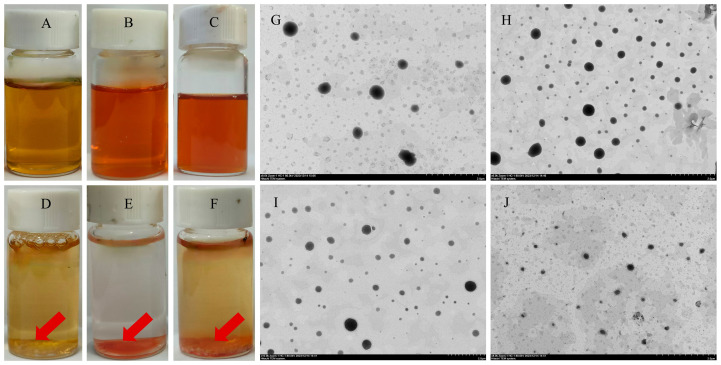
Appearances of TFSs and suspension. (**A**) PTF-TFSs; (**B**) TAN-TFSs; (**C**) PTF/TAN-TFSs; (**D**) PTF-Suspension; (**E**) TAN-Suspension; (**F**) PTF/TAN-Suspension; (**G**) TEM image of KB-TFSs; (**H**) TEM image of PTF-TFSs; (**I**) TEM image of TAN-TFSs; (**J**) TEM image of PTF/TAN-TFSs.

When the clarity of the drug-loaded TFSs groups and the suspension groups were compared (Figure 1), the TFSs formed uniform and clear solutions while the corresponding suspension groups showed visible flocculent precipitates, suggesting that the TFSs had successfully encapsulated components with varying degrees of hydrophobicity. Additionally, TEM was used to describe the vesicles’ structure. Both the drug-loaded TFSs (H–J) and the blank TFSs (G) had a spherical shape with a smooth, rounded appearance, as seen in Figure 1G–J. However, distinct dispersity differences existed across groups: blank and single-drug TFS showed moderate size heterogeneity with minor fragments/aggregates, while PTF/TAN co-loaded TFS possessed far more uniform particle sizes and nearly no aggregates or debris. The synergistic interaction among PTF and TAN inside the TFS structure may be the cause of this. The transfersomes’ palisade layer contains the slightly hydrophobic PTF, but the hydrophobic core’s interior has the strongly hydrophobic TAN [5,8]. The mutual filling of structural voids by these two components reduces particle aggregation and results in superior dispersion of the system, thereby establishing a structural foundation for subsequent improvements in stability and drug delivery performance.

#### 3.1.2. Particle Size Analysis of TFSs Co-Loaded with Components of Different Hydrophobicities

**Figure 2:**
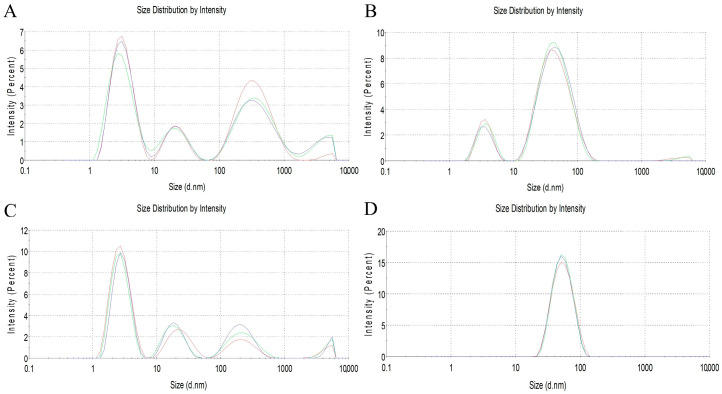
TFSs particle size distribution (x ± s, *n* = 3). (**A**) KB-TFSs; (**B**) PTF-TFSs; (**C**) TAN-TFSs; (**D**) PTF/TAN-TFSs.

According to Figure 2 and Table 1, the co-loaded system PTF/TAN-TFSs had a particle size of 44.52 ± 0.47 nm, which was much larger than the single-loaded systems PTF-TFSs (23.73 ± 0.44 nm) and TAN-TFSs (12.01 ± 6.37 nm). This suggests that the simultaneous co-loading of both components into TFSs increased the particle dimensions. Additionally, the co-loaded system’s polydispersity index (PDI) of 0.22 ± 0.01 was considerably lower than the two single-loaded systems’ PDIs of 0.57 ± 0.003 for PTF-TFSs and 0.33 ± 0.003 for TAN-TFSs. Consistent with the multi-peak profiles observed in Figure 2A,B, the higher PDI values of single-drug loaded TFSs verified their heterogeneous particle populations with multiple size fractions, whereas the unimodal curve of PTF/TAN-TFSs (Figure 2D) confirmed its narrow size distribution. Preliminary findings suggest that the particle size distribution in the co-loaded TFSs system is more uniform, making it less prone to sedimentation and agglomeration, and resulting in high system stability.

#### 3.1.3. Analysis of the Solubilization Effect of Single-Loaded and Co-Loaded TFS Systems

Table 2 illustrates that the single-loaded PTF-TFSs enhanced the solubility of PUE and 3′-HPUE in PTF by 2.20 and 1.44 times, respectively, in comparison to PTF-Suspension. Additionally, the co-loaded PTF/TAN-TFSs showed nearly no solubilization effect on 3′-MPUE in PTF, but they increased the solubility of PUE and 3′-HPUE in the PTF component by 2.27 and 1.76 times, respectively, as compared to PTF/TAN-Suspension. In addition, compared with the documented aqueous solubility of TanIIA (2.8 μg/mL [33]), the solubility of TanIIA in PTF/TAN-TFSs and TAN-TFSs was enhanced by as much as 89.52- and 45.29-fold, respectively. This suggests that the solubilization potential of TFSs for TanIIA was much improved by the addition of the PTF component. When combined, these findings show that TFS may significantly improve the major components’ solubility in a variety of hydrophobic mixes and that co-loading PTF and TAN components improves the solubilization impact even more.

### 3.2. Comparative Evaluation of the Stability of TFSS Co-Loaded with Different Hydrophobic Components

**Figure 3:**
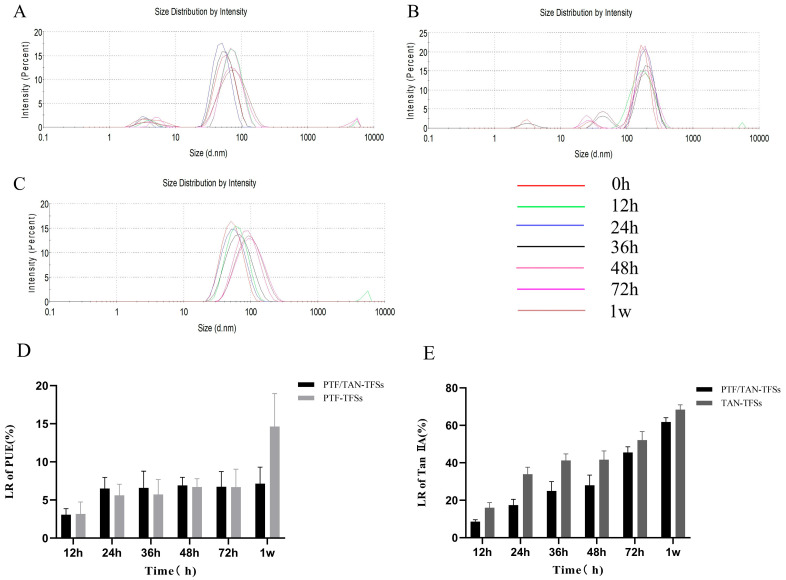
Characterization of TFSs loaded with different components (±s, *n* = 3). (**A**) Particle size distribution of PTF-TFSs; (**B**) Particle size distribution of TAN-TFSs; (**C**) Particle size distribution of PTF/TAN-TFSs; (**D**) Leakage rate (PUF) of TFSs over time; (**E**) Leakage rate (TanIIA) of TFSs over time.

Physical structure and drug-loading stability were the two factors used in this paper to assess the systems’ stability. First, in terms of physical stability, Figure 3 illustrates how the tendency of particle size changes became more noticeable as storage duration increased. The microstructure of the single-loaded TFSs systems (PTF-TFSs and TAN-TFSs) is comparatively loose and prone to deformation, as evidenced by the heterogeneous particle sizes, several peaks in the size distribution, and PDI > 0.3. In contrast, as Figure 3C illustrates, the PTF/TAN-TFSs system’s particle size only started to grow after 48 h; no peak splitting was seen in the particle size distribution, and the PDI stayed below 0.3 the entire time.

This indicates that the structure of the co-loaded TFSs system is comparatively stable and less susceptible to major alterations. Analysis of the leakage rates of single- and co-loaded TFSs systems with PTF and TAN components, as illustrated in Figure 3D,E, showed that after one week, the leakage rate of PUE in PTF-TFSs rose significantly in comparison to the co-loaded PTF/TAN-TFSs (*p* < 0.001). Additionally, the leakage rate of Tan IIA in TAN-TFSs showed a highly significant difference as early as 24 h (*p* < 0.001), whereas the leakage rate of the PTF/TAN-TFSs system did not show a significant rise until 72 h.

### 3.3. Comparative Analysis of the In Vitro Release of Loaded-Component TFSs

**Figure 4:**
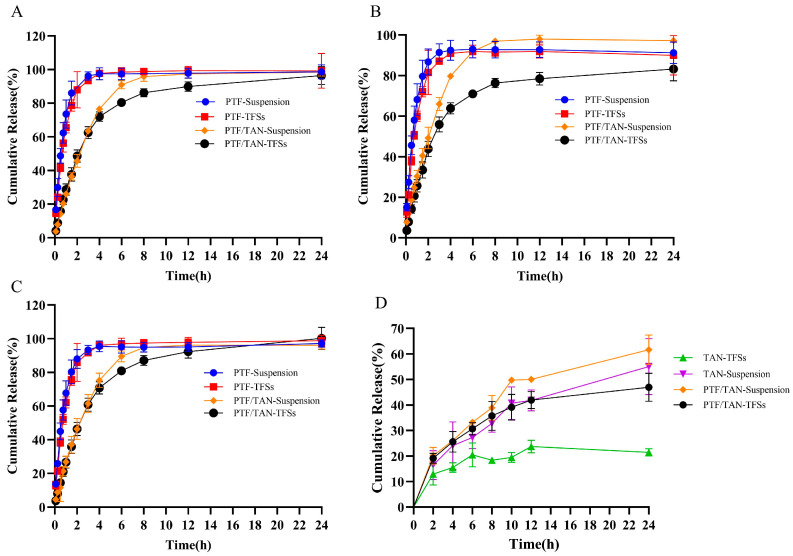
The in vitro release of PUE, 3′-HPUE, 3′-MPUE, and TanIIA in the TFSs and drug suspension. (x ± s, *n* = 3). (**A**) PUE; (**B**) 3′-HPUE; (**C**) 3′-MPUE; (**D**) TanIIA.

As shown in Figure 4, the hydrophilic PTF components PUE, 3′-HPUE, and 3′-MPUE were almost completely released within 6 h in the suspension and within 12 h in the TFSs. In contrast, Tan IIA, as a poorly soluble component of the TAN fraction, exhibited a maximum cumulative release of 62.23% within 24 h in the suspension compared with 46.95% in the TFSs. Preliminary analysis indicated that both the cumulative release rate and the total release of the loaded-component TFSs were slower than those of the raw drug suspension, suggesting that TFSs, as a carrier, can partially delay drug release. Furthermore, the release rates of the single-loaded PTF and TAN systems were both higher than those of the co-loaded system, according to a comparison of single- and co-loaded combinations of components with varying hydrophobicities. This further confirms that the co-loaded TFSs have better drug-loading stability than the single-loaded TFSs. This could be explained by the concurrent dispersion of medications with various hydrophobicities within their individual “comfort zones” [34], which is advantageous for the system’s overall stability.

### 3.4. In Vivo Intestinal Absorption Study of Loaded-Component TFSs

**Figure 5:**
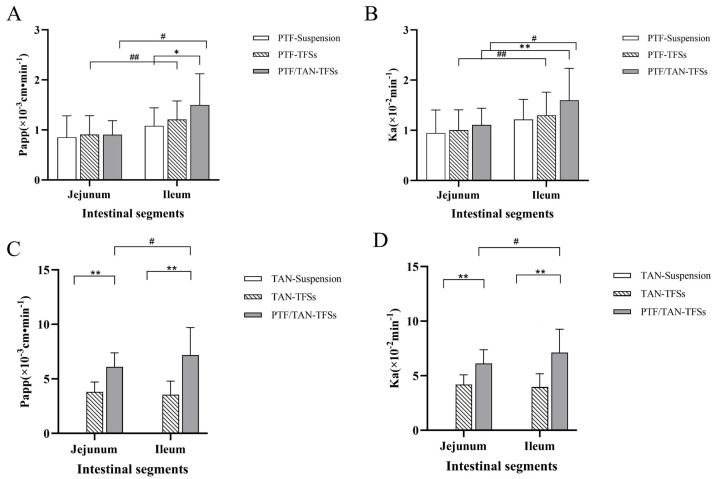
Ka and Papp values of TFSs and suspension in the jejunum and ileum. (x ± s, *n* = 3). (**A**) Papp value of PTF group; (**B**) Ka value of PTF group; (**C**) Papp value of TAN group; (**D**) Ka value of TAN group. Note: Comparison of different formulations within the same intestinal segment in one group: * *p* < 0.05, ** *p* < 0.01; comparison of the same formulation across different intestinal segments between groups: # *p* < 0.05, ## *p* < 0.01.

The ileum is the main site of PUE absorption, as demonstrated by an analysis of Figure 5A,B. The TFSs group’s Papp and Ka values in the jejunum and ileum did not differ significantly from those of the raw drug suspension group. Tan IIA has very low intestinal absorption and is difficult to detect since it is so poorly soluble that it cannot be dissolved in the perfusion fluid by simple physical mixing with the lipophilic TAN components. This is in line with reports in the literature that suggest it is not possible to conduct intestinal absorption tests of TAN components alone [35]. However, as shown in Figure 5C,D, the intestinal absorption characteristics of TanIIA significantly changed after the TAN components were encapsulated into TFSs. The co-encapsulation of TAN and PTF in TFSs could promote the absorption of PUE in the ileal region. The PTF/TAN-TFSs (*p* < 0.01) exhibited a significant absorption-promoting effect in the ileum compared to the duodenum. In addition, as shown in Table 3, in the TFSs group, compared to the TAN single-loading system, the co-loading system increased the Papp and Ka values in the jejunal segment by 1.60-fold and 1.46-fold, respectively, and by 2.02-fold and 1.80-fold in the ileal segment. These findings show that TFSs greatly improve TanIIA intestinal absorption in the ileum and jejunum, while co-loading TAN with PTF further increases TanIIA absorption. This benefit results from two factors: first, a higher concentration of absorbable TanIIA in the intestine is produced by the co-loading system’s stronger solubilization effect; second, a longer intestinal retention time is provided by the co-loading system, which affords an adequate absorption period for TanIIA.

### 3.5. In Vivo Degradation and Distribution Study Following Oral Administration of TFSs

#### 3.5.1. In Vivo Degradation Distribution

**Figure 6:**
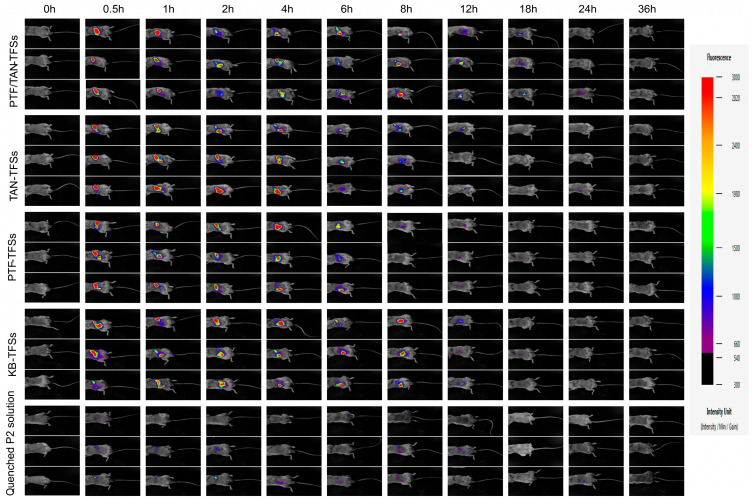
Live images of fluorescence after oral administration of P2-labeled TFSs and quenched P2 solution by gavage in mice. ($\bar{x}$ ± s, *n* = 3).

In recent years, fluorescent bioimaging technology [36] has gradually become the dominant approach for tracking the transport pathways of nanocarriers in in vivo environments and cellular models. Based on the property of P2 probes to aggregate and quench upon contact with water [37,38,39], this paper analyzed the degradation process of TFSs following oral administration: if the vesicular structure of TFSs is disrupted, the P2 probes encapsulated within them are released and come into contact with the aqueous environment, triggering a decay in the fluorescent signal. Figure 6 analysis reveals that the P2 probe solution group, which is quenched, has a very weak fluorescence signal intensity—nearly insignificant—and does not significantly interfere with the later detection of P2-TFSs. On the other hand, the abdomen region of the mice in the P2 probe-labeled TFSs group showed strong and distinct fluorescence signals. Additionally, a strong fluorescence signal persisted for the first one to four hours following delivery. This substantial aggregation induced an initial fluorescence self-quenching phenomenon [40], resulting in a gradual attenuation after 4 h. Over time, the P2-TFSs began to migrate along the gastrointestinal tract, undergoing continuous dissolution and the sustained release of both the drug and the fluorophore molecules, which almost completely disappeared after 18 h. In addition, based on the duration of fluorescence signal retention, using green intensity and above as the threshold, the P2 fluorescence signal in the co-loaded system (PTF/TAN-TFSs group) could be stably maintained up to 18 h, while in the single-loading systems (PTF-TFSs and TAN-TFSs groups), the fluorescence signal was almost undetectable by 12 h. The fluorescence decay rate was significantly faster in the single-loading groups compared to the co-loaded group. These results indicate that P2-TFSs in a co-loaded system have a longer retention time in vivo. It is hypothesized that the distribution of different hydrophobic components in the respective regions of the TFSs enhances their structural stability, thereby protecting more P2 within the hydrophobic regions of the TFSs and prolonging the signal’s retention time in vivo.

#### 3.5.2. Analysis of Retention of Oral Drug Delivery Systems in the Gastrointestinal Tract

**Figure 7:**
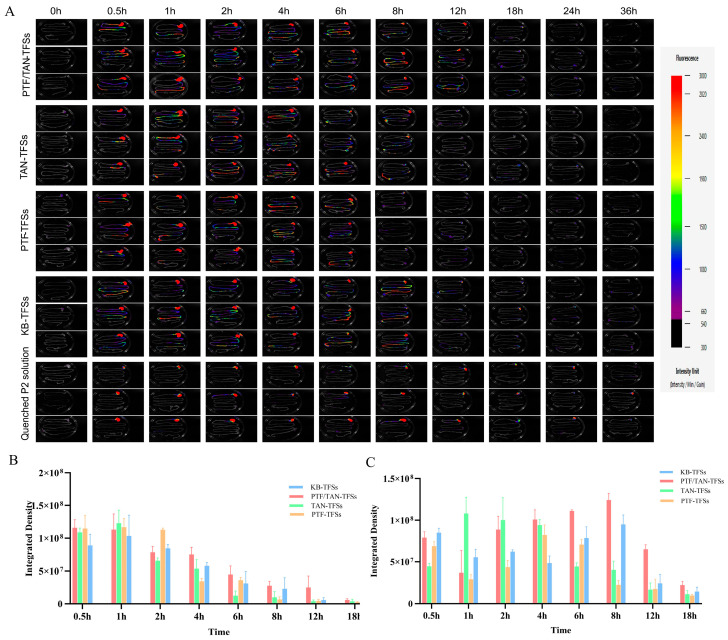
Ex vivo imaging of the whole gastrointestinal tract following oral administration of P2-tagged TFSs groups and quenched P2 solution. (**A**) Ex vivo imaging; Quantification of total fluorescence in (**B**) Stomach; (**C**) Small intestine. ($\bar{x}$ ± s, *n* = 3).

The P2 fluorescent signal retention period of P2-TFSs in the gastrointestinal tract is mostly in line with the in vivo imaging findings, as Figure 7 illustrates. The fluorescence in the P2-labeled TFSs group’s stomach area stayed constant for an hour after treatment. The fluorescence signal rapidly diminished as gastric emptying took place and P2-TFSs gradually decomposed; by eighteen hours, the fluorescence had virtually vanished. Meanwhile, the fluorescence signal in the small intestine increased with gastric emptying and remained strong in the jejunal and ileal segments for up to 8–12 h, indicating that the small intestine is the primary site for the dissolution and absorption of P2-TFSs. Additionally, the fluorescence signals in the cecum and colon suggest that a portion of P2-TFSs was excreted, leading to the loss of fluorescence signals.

The co-loaded PTF/TAN-TFSs systems continued to show strong fluorescence signals in the intestine 12 h after administration, in contrast to the single-loaded PTF-TFSs and TAN-TFSs systems, according to a thorough examination of the variations in the in vivo transport of TFSs with different hydrophobic components. This further demonstrates that the co-loaded system has a longer intestinal retention time with markedly improved stability and persistence in the intestine, which is in line with the in vivo imaging results. This implies that the co-loaded method improves absorption efficiency while lowering medication excretion loss in the colon by increasing the chance of drug–mucosal interaction.

#### 3.5.3. Blood Fluorescence Analysis

**Figure 8:**
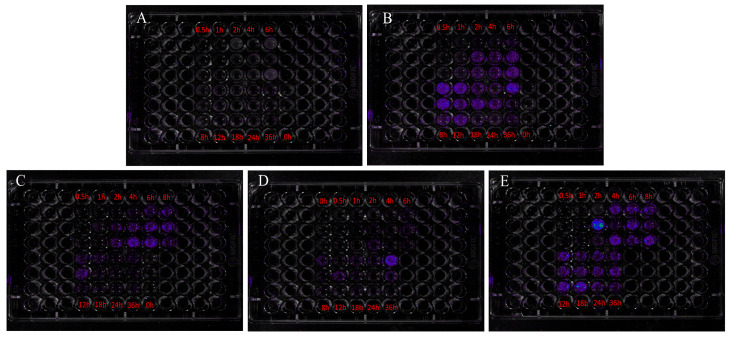
The results of blood fluorescence imaging after oral administration of P2@TFSs and P2 water quenching solution in mice. (**A**) P2 water quenching probe solution; (**B**) KB-TFSs; (**C**) PTF-TFSs; (**D**) TAN-TFSs; (**E**) PTF/TAN-TFSs.

This paper observed the fluorescence signal in the blood after the oral administration of P2-TFSs, as shown in Figure 8. The blank carrier group and the other drug-loaded TFSs groups all had low fluorescence signals, with the exception of the P2 water-quenching probe solution group, which displayed no apparent fluorescence signal. This shows that the probe was effectively encapsulated by TFSs and that the intact TFSs particles were absorbed into the bloodstream. The KB-TFSs group showed stronger signals than the other groups, with fluorescence lasting up to 36 h, according to an analysis of Figure 8B. This shows that the blank TFSs can enter the body as intact TFSs particles and have a greater capacity to encapsulate P2. Additionally, analyzing Figure 8C–E reveals that the PTF-TFSs group showed fluorescence at 4 h, the TAN-TFSs group showed weak blood fluorescence at 2 h, and the PTF/TAN-TFSs group showed fluorescence at 2 h and kept it for 36 h. This difference suggests that the co-loaded system has a longer retention period and a more reasonable time to enter the bloodstream. Its sturdy structure prevents the quick release of drugs in single-loaded systems, which results in abrupt changes in blood drug concentrations. Instead, it provides progressive drug release in the intestine and continuous entrance into the bloodstream. This characteristic is crucial for enhancing drug bioavailability and reducing side effects, and it further confirms the advantage of the co-loaded system in stable drug delivery in vivo.

## 4. Conclusions

This paper’s findings indicate that the delivery method of TFSs can load the strongly hydrophobic tanshinone component TAN and the mildly hydrophobic puerarin flavonoid component PTF at the same time. Among them, the co-loaded system PTF/TAN-TFSs’ particle size, morphology, and structural stability are better than those of the single-loaded systems, suggesting that the co-loaded system’s structural compactness has increased. Regarding the solubilization impact, the TFSs systems can improve the components’ solubility in comparison to the corresponding suspension groups. It is possible to raise the solubility of the strongly hydrophobic Tan II A by up to 89.52 times, whilst the solubility of the mildly hydrophobic PUE can be increased by up to 2.27 times. When weakly hydrophobic components are co-loaded with strongly hydrophobic components, the weakly hydrophobic components are more likely to be distributed deep within the fence layer of the carrier, while the strongly hydrophobic components tend to be distributed within the hydrophobic core of the carrier. It is precisely because drugs exhibit this tendency to distribute within their own suitable regions (i.e., “comfort zones”) [34,41] that more drugs can be encapsulated within TFSs, thereby enhancing the solubilization effect of TFSs on both types of components and providing a viable approach for the synergistic solubilization of multi-component traditional Chinese medicines [42].

Furthermore, both the single-loaded and co-loaded systems demonstrated somewhat greater absorption enhancement than the suspension for the PTF component, but the difference was not statistically significant, according to the absorption trial data from the jejunum and ileum. This is closely connected to the TFSs system’s lack of a noticeable solubilization effect on PTFs. Both the single-loaded and co-loaded delivery methods, however, demonstrated superior absorption improvement for the TAN component compared to the corresponding suspension with the co-loaded system demonstrating a noticeably greater absorption enhancement than the single-loaded system. This outcome is directly linked to the notable improvement in TFSs’ solubilization capacity toward TanIIA with the addition of PTF components, according to the data analysis of the solubilization experiment. TFSs considerably increase the intestinal absorption of TAN, according to additional investigation that combines the degradation behavior, intestinal retention traits, and blood fluorescence signal detection results following the oral administration of TFSs. This also has to do with TFSs’ capacity to pass across cell membranes as whole particles. Moreover, the co-loading system can enhance the structural stability of TFSs, prolong their retention time in the body, and ultimately further improve the drug absorption effect.

Overall, this paper effectively solved the challenge of poor transfersome stability by co-loading components with varying hydrophobicities onto transfersomes. According to this paper, co-loading different hydrophobic components not only makes transfersomes more stable than single-component systems, it also makes the hydrophobic components more soluble, which improves absorption. This sets the basis for future studies into the use of transfersomes in formulations for traditional Chinese medicine. Simultaneously, it offers a research approach for the simultaneous delivery of several components in traditional Chinese medicine while considering the holistic concept of traditional Chinese medicine.

## Figures and Tables

**Table 1:** Particle size and PDI of TFS systems (x ± s, *n* = 3).

Drug Delivery System	Size (nm)	PDI
KB-TFSs	40.88 ± 6.64	0.42 ± 0.055
PTF-TFSs	23.73 ± 0.44	0.57 ± 0.003
TAN-TFSs	12.01 ± 6.37	0.33 ± 0.03
PTF/TAN-TFSs	44.52 ± 0.47	0.22 ± 0.01

**Table 2:** The effect of TFSs on the solubilization of PTF and TAN components. (x ± s, *n* = 3).

Component	Content of Major Flavonoid Components in Kudzu Root (mg/mL)			Content of Major Components in Tanshinone Fraction (ug/mL)
	PUE	3′-HPUE	3′-MPUE	TanIIA
PTF-TFSs	17.56 ± 0.41 **	1.23 ± 0.03 *		-
TAN-TFSs	-	-	-	59.02 ± 9.75
PTF/TAN-TFSs	17.23 ± 0.72 **	1.21 ± 0.05 **	1.97 ± 0.13	186.49 ± 16.83 ^##^
PTF-Suspension	8.36 ± 0.27	0.85 ± 0.02	1.95 ± 0.03	-
PTF/TAN-Suspension	7.94 ± 0.29	0.69 ± 0.03	1.95 ± 0.12	-

Note: Comparison of TFSs groups with corresponding suspensions,* *p* < 0.05, ** *p* < 0.01; Comparison between co-loaded TFSs and single-loaded TFSs groups, ## *p* < 0.01; “-” indicates not detected.

**Table 3:** The Papp and Ka values of TanIIA in jejunum and ileum in in vivo single-pass intestinal perfusion.

	Intestinal Segment	Jejunum		Ileum	
Medication Delivery System		Papp (×10^−3^ cm·min^−1^)	Ka (×10^−2^ min^−1^)	Papp (×10^−3^ cm·min^−1^)	Ka (×10^−2^ min^−1^)
TAN-Suspension		-	-	-	-
TAN-TFSs		3.81 ± 0.90 **	4.18 ± 0.89 **	3.55 ± 1.24 **	3.96 ± 1.22 **
PTF/TAN-TFSs		6.09 ± 1.29 **^△△^	6.12 ± 1.26 **^△△^	7.18 ± 2.53 **^△△^	7.12 ± 2.13 **^△△^

Note: “-”: indicates not detected; Within the same intestinal segment, TFSs compared to suspension, ** *p* < 0.01; Compared to single-load TFSs, co-load TFSs, △△ *p* < 0.01.

## Data Availability

The data presented in this paper are available on request from the corresponding author.

## Abbreviations

The following abbreviations are used in this manuscript:

PTF	Pueraria total flavones
TAN	Tanshinone
PUE	Puerarin
3′-HPUE	3′-hydroxy puerarin
3′-MPUE	3′-methoxy puerarin
TanIIA	Tanshinone IIA
EPC	Egg phosphatidylcholine
SDC	Sodium deoxycholate
TFSs	Transfersomes
KB-TFSs	Blank transfersome
PTF-TFSs	PTF-loaded TFSs
TAN-TFS	TAN-loaded TFSs
PTF/TAN-TFSs	PTF/TAN-loaded TFSs
